# Beneficial and Deleterious Effects of Female Sex Hormones, Oral Contraceptives, and Phytoestrogens by Immunomodulation on the Liver

**DOI:** 10.3390/ijms20194694

**Published:** 2019-09-22

**Authors:** Luis E. Soria-Jasso, Raquel Cariño-Cortés, Víctor Manuel Muñoz-Pérez, Elizabeth Pérez-Hernández, Nury Pérez-Hernández, Eduardo Fernández-Martínez

**Affiliations:** 1 Laboratory of Medicinal Chemistry and Pharmacology, Centro de Investigación en Biología de la Reproducción, Área Académica de Medicina, Instituto de Ciencias de la Salud, Universidad Autónoma del Estado de Hidalgo. Calle Dr. Eliseo Ramírez Ulloa no. 400, Col. Doctores, Pachuca Hidalgo 42090, Mexico; soriajasso@gmail.com (L.E.S.-J.); raquelcarcortes@gmail.com (R.C.-C.); victor9783@hotmail.com (V.M.M.-P.); 2 Hospital de Ortopedia “Dr. Victorio de la Fuente Narváez”, IMSS, Mexico 07760, Mexico; elizabeth.perezh@imss.gob.mx; 3 Programa Institucional de Biomedicina Molecular, Escuela Nacional de Medicina y Homeopatía, Instituto Politécnico Nacional, Mexico 07320, Mexico; nperezh@ipn.mx

**Keywords:** cholestasis, cirrhosis, cytokines, immunomodulation, liver, oral contraceptives, phytoestrogens, sex hormones

## Abstract

The liver is considered the laboratory of the human body because of its many metabolic processes. It accomplishes diverse activities as a mixed gland and is in continuous cross-talk with the endocrine system. Not only do hormones from the gastrointestinal tract that participate in digestion regulate the liver functions, but the sex hormones also exert a strong influence on this sexually dimorphic organ, via their receptors expressed in liver, in both health and disease. Besides, the liver modifies the actions of sex hormones through their metabolism and transport proteins. Given the anatomical position and physiological importance of liver, this organ is evidenced as an immune vigilante that mediates the systemic immune response, and, in turn, the immune system regulates the hepatic functions. Such feedback is performed by cytokines. Pro-inflammatory and anti-inflammatory cytokines are strongly involved in hepatic homeostasis and in pathological states; indeed, female sex hormones, oral contraceptives, and phytoestrogens have immunomodulatory effects in the liver and the whole organism. To analyze the complex and interesting beneficial or deleterious effects of these drugs by their immunomodulatory actions in the liver can provide the basis for either their pharmacological use in therapeutic treatments or to avoid their intake in some diseases.

## 1. Introduction

**Figure 1:**
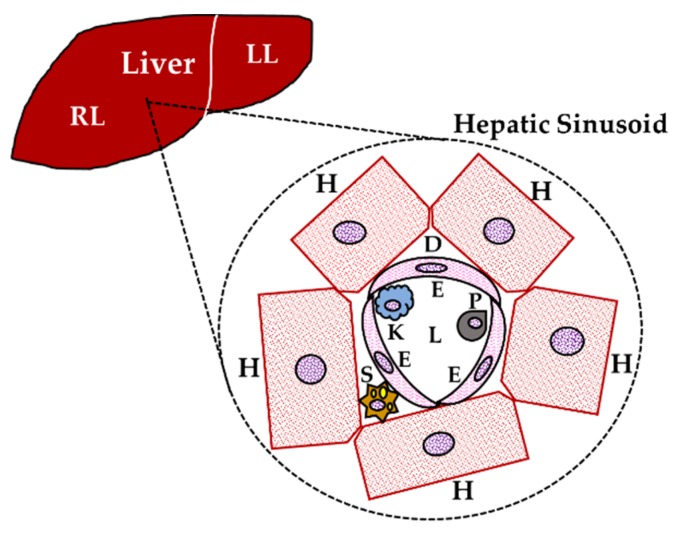
Scheme of the liver and hepatic sinusoid. Liver right lobe (RL), left lobe (LL), and the hepatic sinusoid that is composed by hepatocytes (H), sinusoidal endothelial cells (E), macrophage Kupffer cell (K), natural killer or pit cell (P), Ito or stellate cell (S), space of Disse (D), and lumen (L).

The liver is called the body’s biochemical laboratory because of the many metabolic functions that it accomplishes. Also, the liver is the largest inner organ, with an average weight of 1.5 kg. It is considered a mixed gland: Endocrine because of the insulin-like growth factor 1 (IGF-1) production (among other functional/transport/blood proteins), and exocrine because of the bile production [1,2]. Hepatocytes are the liver parenchyma. They constitute 70%–85% of the hepatic mass that carries out more than 500 metabolic functions [1] (Figure 1). Important hepatic functions include the metabolism of nutrients, storage of vitamins and minerals, production of most of plasma proteins, hormonal balance, and detoxification of compounds produced from metabolism. Also, the liver secretes bile for digestion of lipids by emulsifying fats [1]. The importance of the liver in the metabolism of carbohydrates, proteins, and lipids is fundamental [1]. For instance, the liver regulates the plasma glucose concentration during fasting by glycogenolysis and gluconeogenesis, participates in the synthesis/degradation of proteins, and participates in lipogenesis and synthesis of cholesterol and triglycerides [3,4].

The liver suffers from diverse ailments such as fatty liver (hepatic steatosis), caused by alcoholism, or non-alcoholic fatty liver disease (NAFLD), caused by diabetes and metabolic syndrome (MetS), which in turn may advance to non-alcoholic steatohepatitis (NASH), cholestasis (mechanical or functional stagnation of bile), viral hepatitis (inflammation by infection with hepatitis B or C viruses, (HBV or HCV)), necrosis (non-programmed cellular death by infection or injury), and/or fibrosis (excess of connective tissue). These all may become chronic, which results in the final stage: Cirrhosis. Nonetheless, cirrhosis can advance to hepatocellular carcinoma (HCC), the most frequent cancer of the liver [5]. There are other possible causes of liver damage, such as endocrine disruptors, which are compounds that alter the normal functioning of hormones. Endocrine disruptors can occur naturally, others are synthetic, and they are mainly considered pollutants such as pesticides, industrial chemicals, and pharmaceutical agents. Indeed, there are diverse drugs that induce liver damage, either by long-term and high-dose administration or by dietary and environmental exposure, for example, acetaminophen, cyclosporine, rifampicin, bosentan, glibenclamide, anticoagulants, phenytoin, and herbal products, as well as sex hormones or their metabolites and analogs, including drugs with hormonal side effects such as metoprolol, naproxen, and clofibrate [6,7]. The liver possesses an extraordinary regenerative capacity to keep its cellular architecture and numerous critical functions intact despite the constant insults due uptake, metabolism, and excretion of both endogenous and exogenous compounds, including drugs. Nevertheless, its anatomical position in the digestive tract, high blood irrigation, and intense activity makes the liver extremely susceptible to injury and cancer [8]. Interestingly, cytokines (small glycosylated proteins mainly produced by cells of the immune system but also by many others) and hormones participate in cross-talk to regulate complex processes in both health and disease states.

The importance of sex hormones is undeniable, since they regulate sexual differentiation and reproduction, and they also have a strong influence on the immune system. The latter influences the endocrine system; hence, both regulate metabolism, as will be discussed later [9,10,11]. Besides, oral contraceptives (OCs), which are the synthetic functional analogs of female sex hormones, have become very popular drugs since they were introduced in market for clinical use in the early 1960s, when for the first time an appropriate, cheap, and reliable method for contraception was available [12]. OCs are often administered as a combination of estrogen and progestin (synthetic progestogen), where estrogen suppresses the follicle-stimulating hormone (FSH) to inhibit the selection and emergence of a dominant follicle and subsequent ovulation, and progestin suppresses secretion of the luteinizing hormone (LH) that creates an intolerant environment to sperm [10]. On the other hand, phytoestrogens (PEs) are gaining relevance as dietary supplements because of their potential beneficial properties. They are naturally found in many plant foods. The chemical structure of PE is diverse and includes flavonoids, isoflavonoids, lignans, coumestans, and stilbenes, which modulate estrogen receptor (ER) signaling pathways as well as estrogen functions and metabolism; thus, PEs are also considered endocrine disruptors because they may alter metabolism and the immune response, mainly in the liver, in the same ways that sex hormones, their metabolites, and OCs do [11,13]. The estrogenic potency of most of the PEs is much lower than endogen estrogens, however some of them have significant binding affinity to ERs and also emulate their non-genomic actions [14,15,16].

Therefore, the aim of this review is to provide current information concerning the beneficial and deleterious effects of female sex hormones, OCs, and PEs on the liver by their immunomodulatory properties. The female sex hormones, their metabolites, as well as their synthetic (OC) and natural (PE) functional analogs will be mainly discussed because of their stronger effects on the liver, metabolism, and immune system. Besides, the number of women taking these drugs is much higher than the number of men ingesting them, although as commented above, men are also exposed to endocrine disruptors from the environment.

## 2. Liver and the Immune System: Immunomodulation

### 2.1. Cytokines and the Th1/Th2 Theory

The liver is considered an important immunological organ because of its high population of immune cells; the myeloid cells such as neutrophils, macrophages, and Kupffer cells (the liver resident macrophages); as well as the lymphoid cells such as natural killer (NK), B, and T cells [8]. T lymphocytes produce high concentrations of cytokines, and they present on their surface specific receptors to antigens that recognize exogenous pathogens as well as endogenous cells in autoimmune diseases; thus, T lymphocytes can be divided into two subsets depending on the CD4+ or CD8+ surface molecules or co-receptors expressed on them. CD4+ T lymphocytes are commonly divided into regulatory T cells (Treg) and conventional T helper cells (Th), which in turn can be divided into Th1 and Th2 cells [17,18]. In 1986, it was found that mouse T helper cells expressed two different patterns of cytokines with diverse and opposing functions, they were named Th1 and Th2 cells; then, that theory of immune regulation was adapted to human immunity homeostasis between Th1 and Th2 cell types that directed different immune response pathways, in health and disease, through their two sets of cytokines [19]. It was established that Th1 cells drive the type-1 pathway of cellular immunity to fight viruses, intracellular pathogens, or eliminate cancerous cells. Th2 cells drive the type-2 pathway of humoral immunity, inducing allergic responses and promoting the production of antibodies to combat extracellular organisms (e.g., elimination of parasites); however, excessive activation of either pattern can provoke the establishment of a disease, and either pathway can down-regulate the other one. Nevertheless, the Th1/Th2 theory cannot explain numerous human immune processes because human cytokine networks do not only follow Th1 or Th2 patterns [20]. Th1 cells produce typical cytokines, such as interferon (IFN)-γ, interleukin (IL)-2, and IL-12, while Th2 cells produce IL-4, IL-5, IL-10, and IL-13. Actually, IL-10 possesses the highest down-regulatory activity on Th1 cells [18,19,21]. It has been assumed that Th1 cytokines tend to be pro-inflammatory mediators, which participate in the establishment and perpetuation of several diseases, including liver injury. For instance, IL-2, IL-8, IL-12, IL-17, and IFN-γ are representative pro-inflammatory Th1 cytokines involved in the host defense against bacterial infections in liver [22]. Besides, the most important pro-inflammatory cytokines are tumor necrosis factor (TNF)-α, IL-6 (a bidirectional cytokine, pro-inflammatory preferably), and IL-1β [23,24]. On the other hand, Th2 cytokines principally have anti-inflammatory effects and include IL-1Ra, IL-4, IL-5, IL-13, TGF-β (although this is a pro-fibrogenic cytokine in the liver), IL-13, and, the most important anti-inflammatory cytokine, IL-10 [24,25].

Etiological agents, including drug-induced injuries, cause a disturbance on the normal balance of anti-inflammatory/pro-inflammatory cytokines in the organism, which often exacerbates the pro-inflammatory response and, in consequence, blunts the anti-inflammatory response. Then, if this injury is strong enough during an acute event or is chronically persistent, this deleterious process may lead to disease. Hence, immunomodulation is intended to reinstate the equilibrium of cytokines into the normal homeostatic levels without causing either the depletion of any of them or the exaggerated increase of others. Th1/Th2 immunomodulation is really complex, and it has emerged as an unquestionable pharmacological challenge, particularly for the therapy of diverse illnesses and autoimmune diseases; therefore, this is a current area of research aimed to design immunomodulatory treatments focused on either dampening the overactive responses or reinforcing the weak ones by manipulating the Th1/Th2 balance in diseases [21]. Besides the Th1/Th2 theory for lymphocytes, a dichotomy has been proposed for macrophage activation, the classic M1 and the alternative M2 phenotypes, which have emerged because of several functions and the important immune ligands. M1 is activated by lipopolysaccharide (LPS), IFN-γ, and TNF-α, leading to Th1 responses; while, M2 is subdivided into three groups, M2a (IL-4 and IL-13, Th2 responses), M2b (immune complex, Toll-like receptor (TLR)/IL-1Receptor, Th2 activation), and M2c (IL-10, TGF-β, immunoregulation/tissue remodeling) [26]. Moreover, Kupffer cells display the M1 phenotype during NAFLD, but they switch to the M2 phenotype when the peroxisome proliferator-activated receptor (PPAR)-γ is up-regulated, improving the pro-inflammatory state [27]. Indeed, macrophage activation and responses are regulated by sex hormones in diverse tissues [28]. Toll-like receptors (TLR1–10 in humans) are crucial regulators of innate and adaptive immune responses. They all are present on parenchymal and non-parenchymal liver cells, while TLR2 and 4 are the central mediators of hepatopathies in activating signaling pathways to mainly produce pro-inflammatory cytokines, but also anti-inflammatory ones, to balance the Th1/Th2 ratio [29].

### 2.2. Other Sets of Cytokines: Th9, Th17, and Treg cells in the Liver

Besides Th1 and Th2 cells, there are Th17 and Th9 cells, which are related to immune responses and produce diverse patterns of chemokine receptors to promote cellular recruitment under specific conditions [30]. Some functions of these cells are the recruitment of regulatory T cells, which inhibit antigen-specific effectors allowing the regulation of immune responses, and the reestablishment of immune homeostasis [31,32].

Th17 cells are a subset of Th cells whose functions are mediated by diverse cytokines such as IL-17 and IL-22, among others. Indeed, the IL-17 receptor is practically expressed on all types of liver cells, whilst expression of the IL-22 receptor is limited to hepatocytes and epithelial cells [20]. The differentiation of Th17 occurs during T cell activation by IL-6, TGF-β, IL-1, and IL-2, promoting the expression of the transcription factor RAR-related orphan receptor-γt (ROR-γt) via the activation of the aryl hydrocarbon receptor (AHR) and signal transducer and activator of transcription factor (STAT)-3 [33]. Their deleterious effects are preserved by IL-23. Th17 cells produce IL-17A, IL-17F, and chemokine (C-C motif) ligand 20 (CCL20), which depend on local signals such as IL-22, IFN-γ, or IL-10. Also, they express high levels of chemokine receptors (CCRs) CCR4 and CCR6, although information regarding the signaling for their recruitment to inflamed tissues is scarce. Chronic hepatitis virus infection is a common cause of liver fibrosis that may lead to portal hypertension, hepatic failure, and HCC. Th17 cells are involved in liver fibrosis by activating hepatic stellate cells (HSCs) [31,34,35]. Increased expression of IL-17 has been detected in livers from patients with severe liver fibrosis or cirrhosis. Considering the pro-inflammation function of IL-17 and its close relationship between cancer and inflammation, an increase in hepatic Th17 cells in advanced hepatitis B virus-related liver fibrosis (HBV-LF) might effectively explain the continued inflammation and HCC pathogenesis [36].

Th9 cells mainly produce IL-9, a pro-inflammatory cytokine, which possesses fibrogenic activity by increasing collagen I and III deposition in the liver and triggering lobular hepatitis. IL-9 also promotes an inflammatory response by inducing the recruitment of lymphocytes, neutrophils, and macrophages in portal and parenchymal zones in the liver. In addition, IL-9 deregulates liver antioxidant defenses and promotes hepato-renal dysfunction [34,37,38].

Treg cells express the forkhead transcription factor Foxp3 and are potent suppressors of numerous immune responses. IL-2 is a key factor for homeostasis and in the function of the Treg population, as this cytokine is recognized as a T-cell growth factor. Moreover, the antigen CD25 is highly expressed on the surface of Treg cells acting as the alpha chain of the IL-2 receptor. Treg also expresses other proteins such as cytotoxic T-lymphocyte-associated antigen-4 (CTLA-4) and glucocorticoid-induced TNF-α receptor (GITR) [39,40,41,42]. The phenotype and function of Treg cells may vary into circulatory and intrahepatic compartments depending on whether the intrahepatic microenvironment is hypoxic and whether there are plenty of cytokines and metabolites. In the liver, Treg cells respond to (a) the formation of the T cell receptor (TCR) complex with MHC class II on the antigen-presenting cells (APCs), (b) the interaction of CD28/CTLA-4 receptors on cells with CD80/86 on APCs, and (c) the influence of cytokines produced by APC for activation, survival, and differentiation [43].

### 2.3. Hepatic Cytokines

After birth, the liver remains as a hematopoietic organ. This produces all leucocyte lineages from hematopoietic stem cells, and hepatocytes can produce acute phase proteins in bacterial infections; thus, the liver is a very responsive organ in the first line of host defense by activating Kupffer cells and NK cells in the Th1 response [22]. Given the privileged anatomical and immunological site of the liver, this organ receives antigen-rich blood from the gastrointestinal tract through a network of sinusoids under the surveillance of antigen-presenting cells and lymphocytes; therefore, the liver’s population of innate immune cells is enriched in lymphocytes, NK (they also modulate liver injury by equilibrating the Th1/Th2 cytokines), NKT, and Kupffer cells, which are in contact with antigens presented by endothelial cells, more Kupffer cells, and even the hepatocytes, facilitating the organ’s response to the immune system and vice versa [44]. Hepatocytes markedly activate innate immunity to fight invading microorganisms by producing and releasing diverse innate proteins (including C-reactive protein (CRP)), and they respond to the stimulation of pro-inflammatory cytokines through the activation of the nuclear factor (NF)-κB and STAT-3 [45]. Additionally, dysregulation of these innate proteins of immune system may worsen the chronic liver diseases [8].

These many hepatic functions result from the complex interaction of highly specialized cell types structured in the sinusoid, the surrounding hepatocytes with different non-parenchymal cells of the liver, predominantly sinusoidal endothelial cells, HSCs, Kupffer cells, and lymphocytes [46,47]. Cytokines have received increasing attention as potential diagnostic and prognostic markers for preexisting tissue damage (liver inflammation) and cancer involvement. Damaged hepatocytes and cholangiocytes (parenchymal epithelial cells that line both the intra- and extra-hepatic ducts of the biliary tree) release inflammatory mediators that recruit local leukocytes to the site of injury. In fact, cholangiocytes are involved in epithelial innate immunity, inflammation, and the regenerative process in response to liver injury [48]. The leukocytes amplify the inflammation process through production of pro-inflammatory cytokines such as IL-6, IL-1β, and TNF-α, followed by recruitment of T cells. Two cell types are responsible for extracellular matrix (ECM) deposition in cholestatic disease and act as fibrogenic cells in the liver: Portal myofibroblasts, which are fibroblasts transdifferentiated by TGF-β, and subendothelial HSC, which assumes a myofibroblast-like phenotype when activated by TGF-β. Kupffer cells are intra-hepatic macrophages that, once activated, positively modulate liver fibrosis and stimulate fibrogenic cell activation [25,49]. However, cytokines not only participate in liver damage, but they are also critical promoters of liver regeneration, which is a very complex process regulated by cytokines synthesized and released at the site of damage or migrate to the liver by the circulatory system. Actually, liver regeneration is constituted by three stages: Priming, proliferation, and termination phases. IL-6 and TNF-α promote the priming phase, while TGF-α, the hepatocyte growth factor (HGF), epidermal growth factor (EGF), heparin-binding-EGF (HB-EGF), and their common receptor EGF receptor (EGFR), among others, are involved in the proliferation phase; finally, TGF-β controls the termination phase by its antiproliferative and pro-apoptotic actions [50]. Furthermore, estrogen promotes hepatocyte proliferation via estrogen receptor-α (ERα) activation, and such an action is strongly influenced by IL-6 [51,52]. Taken together, the previous data state that the liver is regulated by the immune system, but also the liver is an immunological organ. The liver’s homeostatic functions in health as well as in disease, either acute or chronic, are the result of a strict balance/imbalance of pro-inflammatory and anti-inflammatory cytokines [8].

### 2.4. Cell Signaling Pathways and Effects of Hepatic IL-6

Despite the great importance of TNF-α [53,54,55], and given the complex network of many cytokines that play substantial roles in the homeostasis and injury of liver, IL-6 is a prominent cytokine because of its bidirectional effects, mainly pro-inflammatory ones, and importance as a mediator in the acute phase response and in chronic hepatic diseases, such as cholestasis, necrosis, and fibrosis, by its interaction with other cytokines. In the initial research on IL-6, its many functions were studied, and each research group named this cytokine differently. Later, it was known that these diverse functions revealed the wide spectrum of target organs for IL-6 [56].

Cellular and tissue injuries by pathogens or diseases may go along with systemic changes, which are reactions to counteract or minimize tissue damage. Those systemic changes are referred to as “acute phase response”, although they are present in both acute and chronic inflammatory circumstances [46]. Acute phase proteins are mediators whose plasma concentrations may increase or decrease by at least 25% during inflammatory states, being called either positive or negative acute phase reactants (APRs), respectively [46]. Alteration of hepatocytes by pro-inflammatory cytokines from immune or other cells are reflected by changes in the levels of APR [57]. The Janus kinase (JAK)/STAT pathways promote the transcription of target genes during the acute phase response, enhancing hepatocyte survival, liver regeneration, and improving the impact of NAFLD [45,47].

IL-6 is the major inducer of hepatic acute phase proteins. It is released from neutrophils, monocytes, and macrophages upon TLR stimulation by LPS and other inducers. Activated myeloid cells synthesize the pro-inflammatory cytokines IL-1 and TNF-α, which may provoke a massive production and release of IL-6 from diverse cells such as endothelial cells and fibroblasts, thus operating as a positive feedforward loop [58]. Hepatic IL-6 induces diverse acute phase proteins such as C-reactive protein, serum amyloid A, fibrinogen, and hepcidin [59]. This cytokine also suppresses the synthesis of albumin, termed a "negative APR", because its levels decrease with inflammation. Combinations of cytokines can have additive [60], inhibitory [61,62], or synergistic effects [63,64], and patterns of cytokine production differ under various inflammatory conditions [47,65].

The role of IL-6-dependent signaling in the liver was mainly attributed to an induction of the acute phase response; nevertheless, many studies show the participation of IL-6 in liver regeneration. Liver regeneration involves hyperplasia of all the cell types of the liver. In humans, hepatocyte replication generally starts within a day of major hepatectomy, and replication of non-parenchymal cells, such as endothelial cells, Kupffer cells, and biliary cells, begins somewhat later. After partial hepatectomy (PH) or after partial parenchymal destruction, the liver is restored to its initial functional mass within a few days, during which a complex array of proliferative and hepatoprotective signaling cascades operate involving cytokines, growth factors, and other paracrine and endocrine agonists. After hepatectomy, the Kupffer cells release TNF-α and IL-6. Increasing their serum levels contributes to the initiation of the cell cycle (in the quiescent liver, almost all hepatocytes are in the G0 phase, and after liver injury, they enter the G1 phase) by binding to their receptors. Upon binding to IL-6 receptor on hepatocytes, IL-6 induces gp130 dimerization and the subsequent dimerization of gp130-associated JAKs, which leads to JAK phosphorylation, followed by activation of the transcription factors STAT-3 and CCAAT/enhancer-binding protein beta (CEBP-β)/nuclear factor-interleukin 6 (NFIL-6). This results in enhanced transcription of a variety of genes that play important roles in protecting against liver injury and promoting liver regeneration. The activation of STAT-3 by IL-6 in sinusoidal endothelial cells may also contribute to the hepatoprotective function of IL-6. Besides, IL-6 may regulate liver fibrosis and inflammation via the activation of STAT-3 in hepatic stellate cells and Kupffer cells, respectively. Although IL-6, IL-10, and IL-22 activate (via STAT-3) similar signaling pathways in the liver, they target different types of liver cells. For example, IL-6R and its signal transducing chain, gp130, are ubiquitously expressed in all types of liver cells, including hepatocytes, Kupffer cells, hepatic stellate cells, and sinusoidal endothelial cells, all of which respond to IL-6 stimulation [66,67,68].

## 3. Female Sex Hormones, Oral Contraceptives, and Phytoestrogens

### 3.1. Estrogen Receptors in the Liver

Estrogens are responsible for the liver production of zona radiata proteins and vitellogenins, estrogen feedback at the level of the brain and pituitary, inhibition of oocyte maturation, and reproductive behavior [69]. The signaling mechanism for estrogens involves binding to ERs either within the cell (intracellular) or on the cell membrane. ERs have been shown to be widespread in the body of several species, but most of the attention has been on the liver because of the importance of estrogen for vitellogenesis. The presence and quantity of ERs in target organs such as the liver and ovary is important in order to interpret the biological roles of estrogens regarding beneficial and deleterious effects of sex hormones in the liver in animal models and humans [70].

The liver is a dimorphic organ and expresses the ERs, ERα and ERβ. Although ERα is highly predominant, both are present on parenchymal and non-parenchymal cells, making the liver responsive to the actions of estrogens [71]. In fact, all immune non-parenchymal cells (monocytes, Kupffer cells, NK, B, and T cells) express ERs [72]. These receptors classically act as transcription factors, traveling between the cytoplasm and nucleus to regulate the expression of numerous genes participating in the cell cycle, proliferation, apoptosis, and inflammation. Lately, the non-classical role of ERs in cell signaling has emerged as a prominent pathway of regulation. Despite ERα and ERβ sharing important characteristics, such as structural homology and ligand binding properties, they function very differently and frequently antagonize each other’s actions; therefore, the ratio of expression of both subtypes produces a significant impact on the final cellular responses to estrogen. Interestingly, male livers express ERα at a significantly higher level than females [73]. The liver diseases associated with OC intake in females and alcoholism in males modify the ER levels in cytosol and nuclei in human livers. In fact, nuclear ER levels are higher in neoplastic hepatic tissues than in normal ones, which suggests a greater responsiveness to estrogens in neoplastic liver tissues [71,74].

### 3.2. Estrogen Receptor-Signaling in the Liver

**Figure 2:**
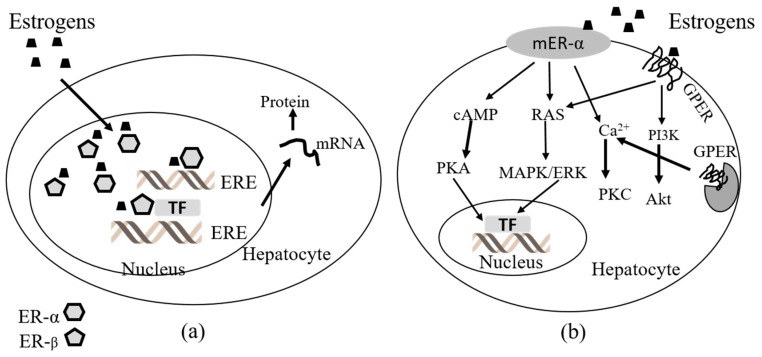
Intracellular pathways of estrogens via estrogen receptors (ERs) in hepatocyte cells. (**a**) Genomic effects of estrogens via nuclear ERs. (**b**) Non-genomic effects of estrogens via membrane-associated ERs. ERE = Estrogen response element; TF = Transcription Factor.

Estrogens act on nuclear ERα and ERβ as well as membrane-bound receptors, including G protein-coupled ER (GPER, also known as GPR 30) and membrane-associated ERα and ERβ variants. All these nuclear and membrane ER subtypes are expressed in the livers of male and female humans and rodents at different levels compared to reproductive organs such as the uterus, ovaries, prostate, testes, and breasts [75,76,77]. ER levels in male and female rat livers have been studied and suggests that the levels of nuclear ERs are not sex dependent but are age dependent, as levels of ERs are similar between male and female rats and vary over the course of life in a comparable manner in males and females [78]. Specifically, ER levels in the liver of male and female rats are the highest during the perinatal period, decline until the onset of puberty, and then increase to reach a post-pubertal peak. Additionally, ER levels are maintained at a stable level across the estrous cycles of female rats [78,79]. Finally, it was reported that the ER concentration in the rat liver increases evidently at puberty [79]. Therefore, the liver ERα may be important in recognizing changes in circulating estradiol (E2) levels, in response to reproductive signals during transitions of different stages of the estrous cycles, and to switch appropriate genetic programs for adaptation of the hepatic metabolism to the energy requirements of each stage. Besides, the hepatic ERα could serve as a peripheral coordinator of energy homeostasis because ERα also exists in the form of membrane-associated receptors. Some evidence showed that full length ERα and truncated ERα may exert actions via non-genomic signaling, which is faster than classic genomic signaling. Commonly, non-genomic signaling involves the activation of intracellular second messenger systems such as protein kinase C (PKC), protein kinase A (PKA), and mitogen activated protein kinase (MAPK) regulated by extracellular signal-regulated kinase (ERK) [80]. Structurally, GPER is different from ERα and ERβ and is a seven-transmembrane domain G protein-coupled receptor located at the cell membrane and endoplasmic reticulum membrane. GPER is able to rapidly activate different non-genomic estrogen signaling pathways including PKA, MAPK/ERK, and phosphoinositide 3-kinase (PI3K) [80,81] (Figure 2).

### 3.3. Sex Hormone Receptors in HCC

Estrogens, progestins, and androgens are lipophilic ligands that bind to transcription factors, which belong to the superfamily of intracellular receptors. They can be activated in the presence of the related ligand or in its absence via post-translational modifications stimulated through the intracellular signaling of membrane receptors, also known as the non-genomic actions [82,83]. Consequently, receptor activation happens via diverse pathways that involve genomic or non-genomic actions [84]. Indeed, in genomic actions the activated receptor may directly bind to the DNA-responsive elements in the gene-specific regulatory regions, or in the non-genomic actions other pathways involved in cell proliferation may be stimulated by interacting with specific proteins in the cytoplasm or in the nucleus [84]. For detailed information about the molecular mechanisms of estrogens and androgens in the gender disparity in HCC, please see the references [85,86].

The progesterone (P4) signaling in HCC has not been explored so deeply. It is well-known that P4, through progesterone receptors (PRs), induces reactive oxygen species (ROS) production and intracellular pathways, resulting in TGF-β1 expression, rat HSC activation, and fibrogenic effects in liver [84]. Further studies are required to prove the possibility that P4 might cause a microenvironment that favors the development of tumors and hepatocarcinogenesis. The effects of progestins on cell signaling pathways without transcription processes depend on conventional PR, suggesting dual functions of PR as both a nuclear transcription factor and a modulator of cell signaling pathways. In humans, the PR-A and PR-B proteins share a sequence containing a polyproline SH3 domain (PPD) interaction motif within the N-terminal domain [84]; hence, the interaction of PR with Schmidt-Ruppin A-2 viral oncogene tyrosine-protein kinase (Src) seems to be a function of the receptor, dissimilar from its transcriptional activity, and it is dissociable because of the point mutations in the PPD interaction motif, such as PR-BΔSH3 [84]. Besides, progestins regulate distinct target genes such as cyclin D1 (CCND1) that lack direct PR binding response elements (PREs) by activation of Src/MAPK signaling. Progestin induction of CCND1 has been assessed in cells that express PR-B but not in PR-A or PR-BΔSH3. On the contrary, the induction by progestins of serum and glucocorticoid regulated kinase (Sgk) genes containing a classical PRE was observed with both PRs as well as PR-BΔSH3. Besides, Src and MAPK inhibitors did not affect the induction suggesting that PR activation of extra-nuclear signaling pathways is important to regulate certain target genes and cell cycle progression [84].

### 3.4. Immunomodulatory Effects of Female Sex Hormones, Oral Contraceptives, and Phytoestrogens in the Liver

Males and females show differences in autoimmune diseases [87], and HCC has a higher prevalence and aggressiveness in males than in females [85]; thus, it is thought that male and female sex hormones have stimulatory and protective effects in the development and progression of diseases. Sex hormones regulate the immune system by their nuclear receptors; thus, they mediate the gene expressions of key proteins and cause gender-specific cellular environments and responses [87]. Estrogens also modulate the production of TNF-α, IL-6, and IL-10 in Kupffer cells by non-genomic actions (likely GPER binding) involving the attenuation of NF-κB, activator protein-1 (AP-1), and mainly MAPK pathways, although to a lesser extent than by ERs-mediated genomic actions [88]. Besides, there are some other non-genomic signaling pathways by which estrogens regulate cytokines and their impact on inflammation, metabolism, and gender-related hepatic functions. Estrogens modulate the growth hormone (GH) actions because of their control on the growth hormone receptor (GHR) expression and intracellular signaling, that is a cytokine receptor of type I without intrinsic kinase activity. Principally, E2 induces the suppressor of cytokine signaling (SOCS)-2, that negatively controls the GHR-JAK-2/STAT-5 pathway; in fact, disturbances of this signaling route are related to hepatic steatosis, fibrosis, and HCC [89].

Therefore, sex hormones are immunomodulatory steroids since they regulate the levels of both pro-inflammatory and anti-inflammatory cytokines, mediating the immune response in several reproductive processes and diverse diseases [10,85,87,90,91,92]. There are some reports dealing with the immunomodulatory effects of OC in liver damage or chronic diseases [93,94]. For instance, OC elicited effects on plasma and hepatic cytokines in rats, emulating the possible events in normal or cholestatic premenopausal women who are administered OC chronically [12]. Also, the effects of estrogens on chronic biliary damage and regeneration have been studied in rats. They were associated with the cytokine production of hepatic IFN-γ and IL-6, which are simulated postmenopausal or oophorectomized women with or without estrogen replacement therapy [93]. Herein, E2 impaired liver functions, in a dual way accelerated both liver damage and healing, as well as significantly changed the cytokine milieu in the cholestatic liver. The in vitro and in vivo effects of E2 and P4 on cytokines have been individually contrasted, generating confusing results. Some studies concluded that neither E2 nor P4, nor synthetic hormones like 17α-ethinylestradiol (EE), influence the production of TNF-α and IL-1β in LPS-stimulated human monocytes [95]. Whereas, other authors used mononuclear cells from patients with chronic hepatitis C to assess the effects of E2 and P4 on the production of TNF-α, IL-1β, IL-8, and macrophage chemotactic protein (MCP)-1. They found that E2 had protective actions by inhibiting pro-inflammatory cytokine production and oxidative stress, while P4 evidenced deleterious effects by enhancing them [94]. On the other hand, P4 has shown immunomodulatory and anti-inflammatory effects in epithelial cells since activation of NF-κB and the consequent production of TNF-α and IL-1β were lowered [92].

**Figure 3:**
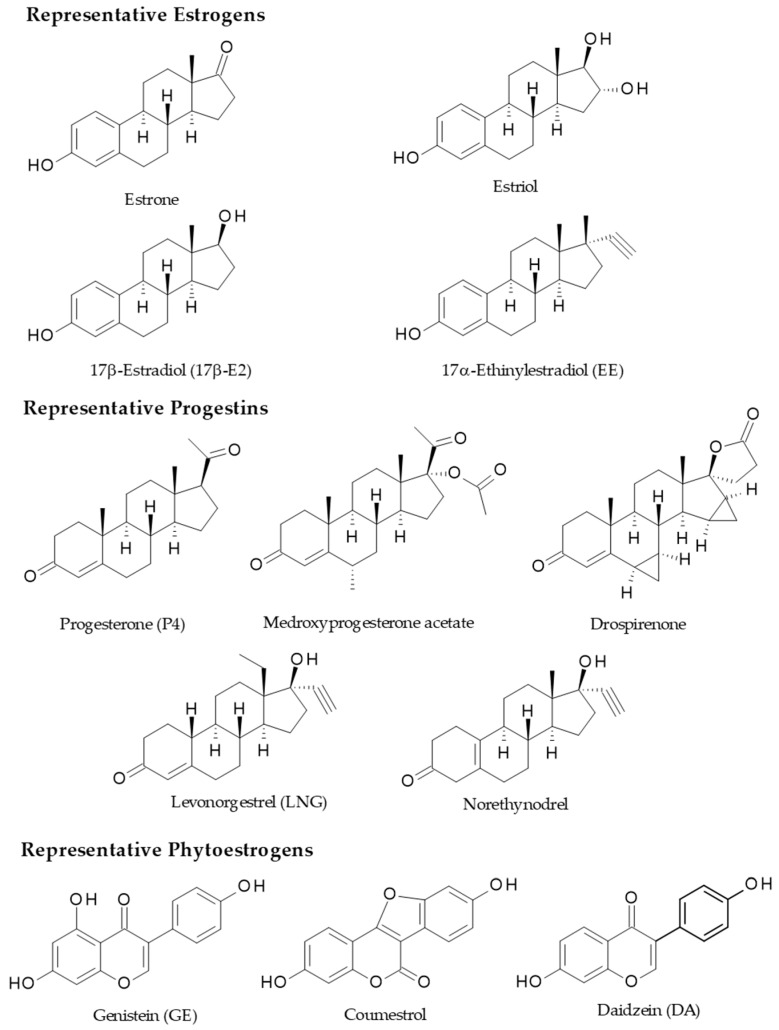
Chemical structure of representative estrogens, progestins, and phytoestrogens.

TNF-α and its antagonist IL-10 are central mediators of liver damage and cirrhosis in both animal models and humans. Indeed, IL-10 down-regulates all the pro-inflammatory, pronecrotic, procholestatic, and profibrotic effects of TNF-α [24,96,97,98,99,100]. Plasma and liver TNF-α from cholestatic rats has been quantified in time course experiments. Some authors observed that plasma concentrations remained unchanged or were faintly augmented compared to normal rats throughout the studies [96,98]; however, others found striking increases in plasma and liver levels [24,96]. Nonetheless, OCs induce remarkable stimulatory actions on plasma and hepatic TNF-α production, which depends on dose and time, proving their immunomodulatory influence on this cytokine [12]. Progestins possess bidirectional effects on cytokines [94,101], and estrogens increase TNF-α synthesis while decreasing IL-10 production in macrophages [90]. The potent inhibitory IL-10 has shown similar plasma and hepatic profiles in plasma and liver similar to TNF-α. Plasma and liver IL-10 was significantly augmented in chronic cholestatic rats, although lower levels of IL-10 have been described in plasma [12,24,96]. Combined OC can up-regulate the cytokine milieu in spite of the high concentrations of IL-10 released to counteract TNF-α and to restore the cytokine imbalance in several diseases [102]. Thus, although OCs promote increased levels of the commented cytokines, chronic liver damage negatively impacts the hepatic amounts of TNF-α and IL-10. This is because they are observed at lower levels than in normal rats, as the injured organ is not capable of producing the same levels of these mediators, which points out the tight relationship between the immune system and the liver in health and disease [8]. Therefore, hepatic functions are mediated by cytokines, which are intensely regulated by sex hormones and their metabolites and analogs (Figure 3) [10,90,95], which in turn regulate inflammation, cholestasis, necrosis, and fibrosis in liver diseases [12,24]. For instance, data from studies on the gender disparity in HCC have unveiled that when estrogens are bound to ERs in the Kupffer cells, they reduce the activity of key pro-inflammatory nuclear factors such as NF-κB, STAT-3, CEBP-β, and the myeloid differentiation primary response protein-88 (MyD88), all of which are promoters of the synthesis and release of IL-6 and IL-1β. Then, the induction of liver damage by these cytokines is inhibited [86]. PE immunomodulatory effects in the liver are commented on throughout the text.

## 4. Effects of Female Sex Hormones, Oral Contraceptives, and Phytoestrogens on the Liver by Immunomodulation

### 4.1. Deleterious Effects by Cholestasis

Cholestasis is defined as an acute or chronic impairment of cholepoiesis (hepatic bile formation) and bile secretion in hepatocytes or ductular cells, both considered intrahepatic cholestasis. In addition, mechanical or functional blocking of the bile flow through intrahepatic or in extrahepatic bile ducts, while bile components come into the blood (bile regurgitation), is considered extrahepatic cholestasis [1,103]; hence, there are two forms of cholestasis by pathogenesis: Obstructive and non-obstructive. Obstructive cholestasis originates from a mechanical obstacle of the bile flow (by inflammation, choledocholithiasis, tumors, and scars), and such bile stagnation affects the ductular cells, diverse hepatic zones, or the entire liver. Non-obstructive cholestasis is considered a multifactorial process by either congenital determinants (Wilson’s disease, familial intrahepatic cholestasis, disorders of bile acids (BAs) biosynthesis, etc.) or by acquired impairment of the bile transport system by etiological causes (alcoholism, drugs, infections, autoimmune conditions, etc.), but even a combination may emerge and provoke the biochemical loss of the hepatocellular ultrastructure with changes in the metabolism of BA. Importantly, BAs are the digestive detergents of lipids, which may eventually degrade the hepatic parenchyma, and they are also metabolic and immune regulators [1]. Chronic cholestasis provokes cytokine-mediated inflammation and connective tissue deposition, leading to biliary fibrosis and to the last stage of biliary cirrhosis [1,24]. Currently, it has been proposed that, beyond the detergent/toxic effects of BA, which can be only achieved at very high concentrations, there are alternative mechanisms. The most plausible is that cholestasis induces the release of BA-induced pro-inflammatory cytokines (TNF-α, chemokine CCL2, chemokine C-X-C motif ligand 2, and CXCL2, among others) by hepatocytes in addition to the responses of cholangiocytes and the innate immune cells in liver. Also, it has been observed that only hepatocytes respond to BA during cholestasis, while non-parenchymal cells do not [104].

The liver is a target organ of OC, and old reports point out a higher incidence of hepatic adenoma and HCC correlated to OC intake. Besides, OC users showed a higher incidence of gallstones because of an estrogen-induced altered ratio between cholesterol and bile acids in bile that lowered the cholesterol solubility [74,105,106]. EE causes intrahepatic cholestasis. Indeed, this drug is used as a pharmacological inducer in murine models of intrahepatic cholestasis, promotes biliary excretion of canalicular membrane enzymes [107], and disrupts the integrity of the hepatic tight junction [108]. Women with genetic alterations in bile canalicular membrane transporters are more prone to OC-induced cholestasis because they have a deficiency of the MDR-3 protein [109]. Estrogens are pro-cholestatic agents, for instance E2, but also the progestins, such as norgestrel, increase the cholesterol saturation of bile and, in turn, promote gallstones [110]. Estrogens increase the sensitivity of Kupffer cells (chiefly producers of pro-inflammatory cytokines) to the hepatotoxic endotoxin [111] and, in consequence, the OC may worsen liver damage in alcoholic women with endotoxemia [112]. In fact, LPS is a cell wall constituent of gram-negative bacteria. This is a highly conserved inducer of pro-inflammatory cytokines (e.g., TNF-α, IL-6, and IL-1β) released from Kupffer cells, activated sinusoidal cells, and cholangiocytes, which reduce the expression and the function of hepatocellular and cholangiocellular transporters and cause cholestasis as well as oxidative, mitochondrial, and endoplasmic reticulum stress [113,114]. Besides, the pro-inflammatory cytokines differently regulate hepatic transporters depending on the mechanism of cholestasis [97]; indeed, TNF-α and IL-1β downregulate the bile salt export pump (BSEP) and decrease BAs secretion [115].

EE administration reduces the bile flow and produces impairment of transport mechanisms in both basolateral and canalicular hepatocyte membranes; consequently, biliary excretion of BA, bilirubins, cholesterol, phospholipids, and HCO_3_^−^ are reduced. In murine models, EE treatment decreases sinusoidal membrane surface density and causes a deficiency in sinusoidal transporters, which perform the uptake of cholephilic compounds [103]. Also, cholestatic damage may be induced either subchronically in a few days by administration of EE or within minutes by the procholestatic metabolite estradiol-17β-glucuronide (E2-17G) [116]. The uptake of sex hormones and OC metabolites from the sinusoidal blood into the hepatocytes requires organic anion transporting proteins (OATPs). Then, sex hormones, OCs, and metabolites can exert regulatory/metabolic actions or are further metabolized into hepatocytes, but eventually they must be transported into the canalicular lumen by the basolateral multidrug resistance-associated protein (MRP)-2 to be excreted. Once they have been transported to the luminal side, they may provoke cholestasis. In fact, E2-17G, EE, and P4 metabolites are called trans-inhibitors of the BSEP, since they exert their inhibitory effect on the transporter from the canalicular lumen, which causes a stagnation of toxic BAs into the hepatocytes as well as liver damage by inducing pro-inflammatory cytokines release, as commented above [104,117]. Besides, E2-17G induces a cholestatic mechanism through transporter internalization, wherein there are two phases. The first cholestatic phase is the endocytic internalization of MRP-2 and BSEP; later, during the second phase, a recovery happens with the concomitant and spontaneous re-insertion of subapical vesicles in the canalicular membrane. When a repeated dose of E2-17G is administered to rats, two processes are elicited: A deeper internalization of MRP-2 and an abnormal localization of a reduced fraction to the lateral membrane [118].

EE-induced cholestasis causes a small modification of BSEP expression, while MRP-2 expression is highly altered at a post-transcriptional level. Besides, EE produces a transcriptional down-regulation on the Na^+^-taurocholate cotransporter protein (NTCP) and OATPs, but it conversely up-regulates MRP-3 too. It has been suggested that the nuclear ER-α mediates EE-induced cholestasis and the alterations in transporter expression [119]. Sex hormones, particularly estrogens, regulate the proliferation and function of cholangiocytes during cholestasis, as a repairing and compensatory mechanism, to equilibrate the loss of impaired bile ducts. Also, cytokines such as TNF-α and IL-6 from LPS-stimulated Kupffer cells induce cholangiocyte proliferation [120,121]. Therefore, sex hormones, OC, or their metabolites, may influence the cytokine milieu by immune cells during liver damage [9], as is the case of LPS-induced cholestasis that activates hepatocytes to release pro-inflammatory cytokines, which in turn activate the Kupffer cells to promote liver damage, or even the very same drug-induced cholestatic process induced by these hormones or their metabolites; thus, sex hormones may act by direct and indirect immunomodulatory mechanisms to provoke or regulate cholestasis. On the other hand, PE resveratrol and ginsenosides have been surprisingly reported as anti-cholestatic agents in the EE murine model, evidenced by reduced levels of biochemical markers of cholestasis, oxidative stress, as well as of the pro-inflammatory cytokines TNF-α, IL-6, and IL-1β [122,123]. However, there are also PEs that may be pro-cholestatic agents, as in the case of miroestrol and deoxymiroestrol. Such compounds reduce the hepatic expression of BSEP and MRP2 mRNA in mice, either males or females, which may result in intrahepatic cholestasis [124]. Whether these PE regulate transporter expression by immunomodulatory effects is not known.

Progestins are also procholestatic, as evidenced during intrahepatic cholestasis of pregnancy (ICP), wherein the P4 metabolites are crucial in this pathogenesis. Patients with IPC excrete more 3α-hydroxysteroids (5α-pregnan-3α-ol-one (PM4)) and high quantities of mono and disulfated metabolites of P4 (sulfated PM4, PM4-Sul, and epiallopregnanolone-sulfate (PM5-Sul)) in urine. Consequently, excessive amounts of these metabolites in urine in IPC patients has been correlated with failure of biliary canalicular transporters, since they secrete these metabolites from hepatocytes into bile [125]. BSEP, MRP-2, and MRP-3 are altered by the progestin metabolites; moreover, the mixture of PM4-Sul and E2-17G diminish the bile flow and the BA output because of an important non-competitive trans-inhibition of BSEP-mediated BA efflux [125]. Sulfated metabolites of P4 reduce NTCP function in a dose-dependent manner [126]. Currently, it has been proposed that during ICP the marked increase of E2 and P4 (also their metabolites) provoke cholestasis that negatively modify the immune balance by enhancing the activity of Th1/Th17 cells which release pro-inflammatory cytokines (TNF-α, IFN-γ, IL-17, and IL-6), while decreasing the activity of Th2/Treg cells which produce anti-inflammatory cytokines (IL-10 and IL-4), thus the outcome is multiple organ injury, the comprehensive review by Larson et al. [115] is recommended.

### 4.2. Bidirectional Effects on Hepatic Oxidative Stress and Metabolism Regulation

Oxidative stress is the imbalance between oxidative species, ROS and other free radicals, and the protective antioxidants (enzymatic and nonenzymatic compounds)—these latter decrease in the organism [127]; thus, the final sum of oxidative agents and antioxidant defenses yields either homeostatic or deleterious results. The formation of ROS and other free radicals is normal during a healthy state. Actually, free radicals can regulate intracellular signal transduction pathways and gene expressions, occasioning a low cytokine production essential for metabolism and inflammatory processes. Nevertheless, in liver diseases, excessive oxidative stress causes the progression of pathological states and serves as a prognostic sign [128]. Secondary biliary cirrhosis provokes lipid peroxidation (LP), which is a worthy marker of liver damage induced by oxidative stress; in fact, in chronic cholestatic rats, LP promotes a cytokine imbalance [24,96]. Normal and cholestatic female rats administered with OC (EE and norgestrel) showed an increased level of LP. Also, the augmented plasma and liver concentrations of the pro-inflammatory TNF-α were correlated with OC administration [12].

Elevated peroxidation markers have been determined in women aged 40–48 years who used OC, which indicate oxidative stress; also, OC users showed a marked augmentation of LP when compared to non-users and intrauterine users of hormonal and copper devices. In addition, their antioxidants in plasma were significantly diminished [127]. Oxidative stress was measured in women 18–35 years old who consumed an OC combination of EE and drospirenone. Again, lipid peroxides and oxidized low-density lipoproteins (LDLs) were found highly elevated, while the antioxidants β-carotene and zinc were lowered [129]. OCs inhibit antioxidant enzymes in mice; for instance, liver paraoxonase (PON) activity is significantly decreased because of diverse combinations of OC, whilst serum PON activity is augmented. Moreover, the catalase (CAT) activity of erythrocytes is considerably reduced by all OCs tried.

Prooxidant effects of estrogens have been evidenced from both in vitro and in vivo studies. Even a low dose of EE increases hepatic LP and decreases the activities of superoxide dismutase (SOD) and CAT. Besides, it has been mentioned that estrogens or synthetic analogs are activated in the liver; for example, CYP1A produces hydroxylated metabolites of E2 and EE, which can afford catechol E2 or EE metabolites. These in turn generate reactive quinones, by redox reactions, and lastly free radicals, ROS, and mitochondrial damage, ultimately leading to cytokine-mediated inflammation [130]. Conversely, most studies point out that estrogens (primarily E2) possess antioxidant properties by diminishing ROS, the activation of NF-κB, and AP-1. This in turn down-regulates the synthesis and release of pro-inflammatory cytokines as well as up-regulates the production of antioxidant enzymes and induces the expression of the Bcl-2 family proteins; therefore, LP and TNF-α-induced apoptosis are diminished [131,132]. Indeed, ERα and ERβ mediate many of these effects [133]. On the other hand, P4 and progestins, such as norgestrel, possess prooxidant and pro-inflammatory effects, as opposed to estrogens, which may also play an important role in oxidative stress and LP [134,135,136]. However, the progestin norethisterone acetate has been evidenced to possess antioxidant effects. Its administration decreased liver LP as well as increased the SOD and CAT activities in female rats [137]. It is suggested that many of the prooxidant effects of OC are related to the combination of estrogens and progestins used, besides the dose and chronic administration that may elicit cholestatic events and the subsequent retention of high concentrations of BA in hepatocytes. Furthermore, the formation of prooxidant reactive metabolites of estrogens and progestins in the liver contribute to injury, as well as the induction of pro-inflammatory cytokines by themselves, as immunomodulatory agents and in response to cholestasis. Regarding PE, there are reports about the antioxidant effects of resveratrol (as a polyphenol, this is a free radical scavenger) and ginsenosides, which reduce liver peroxidation and pro-inflammatory cytokines and consequently elevate enzymatic antioxidant defenses [122,123].

The liver is the main metabolic organ that carries out both lipometabolism and glucometabolism; therefore, if there are disorders in lipid and/or carbohydrate regulation, they negatively impact the health of the liver. Conversely, both acute and chronic liver injury can aggravate metabolic diseases as well as its own pathological status [4]. The liver and steroid hormones maintain body homeostasis, and the dysregulation of either may lead to liver and endocrine illnesses. Besides, the liver controls steroid hormones, including sex hormones, OC, and PE, by cholesterol metabolism, hormone synthesis enzymes, degradation, release, and steroid carrier proteins. On the other hand, sex hormones, or analogs, actively participate in liver diseases related to MetS; for instance, low levels of androgens and estrogens may predispose to or worsen type 2 diabetes, dyslipidemia, NAFLD, NASH, and HCC, all of which are mediated by hepatic metabolic regulators such as cytokines, androgens, ERs, as well as transcription factors as the novel p53 factor [138].

Beyond its canonical role in reproductive development and function, estrogens also play a role in regulating non-reproductive systems such as immune function, growth, neuronal function, and metabolism [130]. Mice with aromatase deficiency and an inability to synthesize estrogens exhibit disrupted metabolic function [131], and there are dramatic metabolic changes that occur with the normal changes in reproductive status across the lifespan, including during puberty [132], the menstrual cycle [133], and menopause [134]. Additionally, hypogonadism in men is associated with increased risk of type 2 diabetes and metabolic syndrome [135]. Therefore, it is necessary to have a better understanding of the mechanisms underlying the regulation of glucose and lipid metabolism by gonadal steroids [130,135]. Cellular 17β-E2 signaling is mediated mainly via ERα (ESR1) and ERβ (ESR2), although recent findings have demonstrated E2 action via the cell surface G-protein coupled receptor, GPER. ESR1 and GPER are the major ERs expressed in the liver, with ESR1 being much more abundant than GPER [136]. Impaired ESR1 function is associated with obesity and metabolic dysfunction in humans [137,138] and rodents [139,140,141,142]. However, the mechanisms underlying these disorders remain largely unknown.

Some combinations of estrogens and progestins cause ultrastructural lesions in liver and impair diverse metabolic routes, such as protein biosynthesis, energy production, and augmentation of cellular catabolism. Again, these modifications are correlated with the dose and period of OC treatment [139]. Liver glycogen is the main source of energy in the body because glucose is released from the liver when required; thus, the control of the synthesis and degradation of glycogen is vital to regulate plasma glucose levels. The pro-inflammatory cytokines TNF-α, IL-1β, and IFN-γ as well as NO control glycogen synthesis and glycogenolysis; moreover, elevated TNF-α can suppress the glucose uptake [24,140]. Cholestasis exhausts the liver glycogen [24,141]. There are reports about the effects on hepatic glycogen by sex hormones and OC (from the latter, either progestin and estrogen in combination or administered alone). Some authors observed that combined OC increases the hepatic glycogen stores in normal rats [142], which also happened by administering the mixture of EE and norgestrel, although this combination provoked hepatic necrosis [143]. Old references mention that the estrogenic constituents of OC are suspected of being activators of glycogen synthesis [144]. More recently, the hepatoprotective effects of E2 have been shown in a model of diabetes because of their antioxidant properties and by lowering the plasma glucose and increasing the hepatic glucose uptake that augments glycogen in the liver [145]. In a marked divergence, progestins antagonize estrogenic effects [142]; however, levonorgestrel (LNG) affords ambiguous effects: Either it prevents glycogen depletion during liver damage or reduces it in normal rats [146]. Furthermore, in a recent study, administration of a combination of EE and norgestrel did not have any impact on the hepatic glycogen levels in both normal and cholestatic female rats [12]. Hence, not only do estrogens and progestins act as opposing driving forces on liver glycogen metabolism, or lipid and carbohydrate metabolism, but androgens and estrogens also produce divergent effects on diverse metabolic diseases such as NAFLD-induced HCC [147].

The PE biochanin A (BCA) was proven against fibrotic complications induced by CCl_4_, protecting it from oxidative stress measured by LPO, GSH, SOD, total antioxidant content, catalase activity, and inhibited biomarkers of NO inflammation and its inducible synthase (iNOS), cyclooxygenase (COX)-2, and CD45 expression [148]. Other studies evaluated BCA as an antifibrotic agent and revealed the inhibition on NF-κB, TGF-β1, MMP9, and TNFα by BCA [149]. Also, BCA exerts hepatoprotective effects during treatment with ritonavir through modulating oxidative stress, inflammation, and apoptosis; reversing tissue degeneration induced by Bax (also known as bcl-2-like protein 4), caspase-3, NFκB and eNOS activation; and persuading the Bcl_2_ and pAkt (protein Kinase B) levels in both hepatic tissue and serum. This suggests its therapeutic role in hepatotoxicity caused in retroviral treatment [150]. Moreover, BCA protected against LPS/galactosamine (GalN)-induced acute liver injury in mice by activating the nuclear factor erythroid 2 (Nrf2) pathway and inhibiting NOD-like receptor 3 (NLRP3) inflammasome activation [151]. Also, BCA increases the expression of hepatic PPAR-α and its regulatory proteins, promoting the recovery of metabolites involved in phosphatidylcholine synthesis, lipogenesis, and β-oxidation in the livers of obese mice [152]. Thus, BCA may be a potential therapeutic agent for the prevention of obesity-mediated hepatic steatosis and insulin resistance.

Resveratrol (RSV) is a phytoestrogen that protects against atherosclerosis and hepatic steatosis. RSV attenuates steatosis and proprotein convertase subtilisin/kexin type 9 (PCSK9) expression through down-regulation of sterol regulatory element-binding protein 1 (SREBP-1c) expression, at least in part through ERα-mediated pathway in L02cells [153]. RSV has been proven to induce a mitochondrial complex I-dependent increase in NADH oxidation, causing the activation of sirtuin in hepatocytes. Inclusively, this finding was observed with in vitro experimental models of isolated enzymes and HepG2 cells treated with RSV and with an in vivo aging model of mice fed with RSV [154].

Genistein (GE) has a beneficial effect on NAFLD. These benefits are believed to be associated with energy metabolism, antioxidation, anti-inflammation, and antifibrosis in the prevention of NAFLD-related liver tumorigenesis [155]. Numerous studies have investigated the effects of GE in liver cancer cells in vitro, and results show that genistein inhibits the growth of HepG2 and Hep3B cells and suggest that genistein is an effective isoflavonoid that induces apoptotic signaling [156,157]. Studies on the long-term effects of GE in a model of HCC establishment and development in female mice found that GE suppresses cancer initiation and development through increasing AMP-activated protein kinase (AMPK) levels in the liver. And in in vitro cultures of Hep3B and Raw 264.7 cells, GE reduced NF-κB levels and down-regulated TNF and IL-6 [158]. GE ameliorates NAFLD in mice and rats, directly targeting COX-1 activity as well as its downstream TXA_2_ biosynthesis, while the TXA_2_ pathway might mediate NAFLD progression by impairing insulin sensitivity [159]. GE can improve hepatic steatosis via AMPK, thus promoting fatty acid oxidation and inhibiting hepatic lipid synthesis in rats treated with a high-fat and high-sucrose diet [160]. GE pre-treatment significantly reduces the increased levels of iNOS and COX-2, consequently lowering the respective NO and prostaglandin (PG)-E2 levels. GE markedly inhibits the production of D-GalN-induced pro-inflammatory cytokines TNF-α and IL-1β. These effects were associated with the inhibition of NF-κB activation, IKKα/β, and MAPK phosphorylation [161,162]. GE alleviates hepatic damage induced by chronic alcohol administration as measured by the antioxidant (HO-1, SOD, CAT, GSH, and GSH-Px), anti-inflammatory (NF-κB, COX-2, TGF-β1, MCP-1, TNF-α, and IL-6) status, or anti-apoptotic (caspase-3) mechanisms in mice [163]. In a model of liver fibrosis induced by CCl_4_, GE induced a significant reduction in the levels of TNF-α and PDGF-BB. In addition, GE had positive effects on the oxidant/antioxidant status and on liver necrosis and fibrosis scores [164]. For a comprehensive and thorough review of the experimental and clinical effects of GE, the review by Wang et al. [165] is recommended.

GE and daidzein (DA) supplements also improved the plasma total cholesterol, triglycerides, HDL-cholesterol/total cholesterol, free fatty acids, and hepatic triglyceride concentrations in C57BL/KsJ-db/db mice, therefore exerting anti-diabetic effects in type 2 diabetic conditions by enhancing glucose and lipid metabolism [166]. Overall, another study indicated that DA potently induced apoptosis of SK-HEP-1 hepatic cancer cells via a mitochondrial pathway that was correlated with the up-regulation of Bak and down-regulation of Bcl-2 and Bcl-xL proteins. Besides, DA enhanced the release of mitochondrial CYP and the activation of pro-apoptotic APAF-1, caspase 9, and caspase 3 [167].

Supplementation with enterolactone (ENL) in HepG2 cells showed a shift in the ω-6/ω-3 balance towards ω-6, as well as the increase in COX-2 and TNFα protein expressions [168], which led to the development of hepatic insulin resistance (IR) [169]. This consequence may be the result of an elevated intracellular ceramide accumulation caused by an increase in the de novo synthesis pathway, which led to enhanced apoptosis of HepG2 cells [169]. Additionally, the increase in intracellular ceramide content in cultures of primary rat hepatocytes is due to the pivotal role of transporters in facilitating fatty acid influx (FATP2), accumulation of ceramides (CERT), and export to the media (MTP and ABCA1) [170].

The studies about xanthohumol (XN) and 8-prenylnaringenin (8PN) indicated that both improved metabolic markers and the cholestasis indicator alkaline phosphatase levels in mice demonstrated that treatment with both PEs resulted in activation of the AMPK signaling pathway, thus suppressing lipogenesis [171]. These results agree with findings showing that XN attenuates atherosclerosis in apolipoprotein-E-deficient (ApoE^−^/^−^) mice fed a Western-type diet. XN decreased the levels of hepatic triglycerides and cholesterol, activated AMPK phosphorylation, and inactivated acetyl-CoA carboxylase, which also reduced the expressions of the mature sterol regulatory element-binding protein (SREBP)-2 and SREBP-1c mRNA and pointed out a reduced hepatic lipogenesis in mice. Also, XN promoted the concomitant induction of hepatic mRNA expression of carnitine palmitoyltransferase-1a in ApoE^−^/^−^ mice, which suggests an elevated fatty acid beta-oxidation [172]. XN has been shown to have hepatic fibrosis-inhibiting activity on primary human hepatic stellate cells and hepatocytes in vitro, and these effects of XN on hepatic inflammation and fibrogenesis were studied in a murine NASH model. XN inhibited the activation of primary human HSCs and induced apoptosis in activated HSCs in vitro in a dose-dependent manner. In contrast, XN doses as high as 50 mM did not impair viability of primary human hepatocytes. However, in both cell types, XN inhibited activation of the transcription factor NF-κB and expression of NF-κB-dependent pro-inflammatory genes. In a murine model of NASH, XN feedings reduced hepatic inflammation and the expression of profibrogenic genes [173]. Potentially, XN may target fibrosis, cirrhosis, and HCC associated with chronic HCV infection because of the anti-fibrotic effects, brought on by the inhibition of hepatic TGF-β1 expression, and because of XN-induced inhibition of NF-κB-mediated inflammation, cancer, and the reduction of hepatic inflammation, steatosis, and fibrosis in HCV-infected Tupaias [174].

### 4.3. Beneficial Effects on Hepatic Fibrosis

Fibrosis is the excessive production and deposition of collagen and other extracellular matrix components, because of increased fibrogenesis and lowered fibrolysis, in the course of chronic hepatobiliary diseases, which lead to the final stage cirrhosis [175,176]. Fibrosis is a complex process orchestrated by the production and release of pro-inflammatory and/or pro-fibrotic cytokines such as TNF-α, transforming growth factor (TGF)-β (but frequently anti-inflammatory), platelet-derived growth factor (PDFG), IL-1β, and IL-17 as well as those anti-fibrotic cytokines which might counterbalance the final result such as IL-10, IL-22, IL-6, and IFN-β/γ (IL-6 and IFN-γ are generally pro-inflammatory) [177]. Consequently, the pro-fibrotic cytokines virtually activate all liver cells, mainly the Kupffer cells, that are an important source of more pro-inflammatory and/or pro-fibrotic cytokines, which amplify and aggravate the fibrogenic process. As a result, the HSC are activated since they are the central target and mediators of liver fibrosis. In turn, activation of NF-κB is prompted by triggering the production of the major pro-fibrotic cytokine TGF-β, which leads to chronic fibrosis and cirrhosis [178,179,180]. Interestingly, HSC in male and female rats are ERβ-positive cells that do not express the ERα [181].

Excessive accumulation of hepatic collagen is observed in cholestasis-induced cirrhotic rats [24,98]. Besides, it was reported that E2 enhances the LP level, collagen amount, and cirrhosis degree induced by thioacetamide [182]. Nevertheless, OC administration reduces liver fibrosis in cholestatic, cirrhotic female rats despite elevated plasma and hepatic TGF-β levels [12]. In fact, several reports have demonstrated that combined OC as well as the estrogens E2 and EE diminish collagen synthesis in different organs in a dose-dependent manner, either in vitro or in women. It has been proven that estrogens modulate the TGF-β/Smad-2 transcription factor signaling pathway. Furthermore, E2 diminished TGF-β production, collagen synthesis, and oxidative stress in various liver injury models in rats [183,184,185,186]. Evidence supports the idea that estrogens act as anti-fibrotic agents by inhibiting the activation of transcription factors as antioxidants as well as the MAPK pathways. Besides, estrogens inactivate the downstream transcription cascade in TGF-β1 expression and HSC activation, while progestins counteract those protective effects, predominantly the P4 [135,187]. The administration of estrogens has beneficial anti-fibrotic effects in female patients chronically infected with HBV or HCV [133,188,189,190]. Chronic liver damage by cholestasis highly increases the plasma and hepatic levels of TGF-β [96,98], which is synergized by combined OC administration [12]. Also, the treatment of normal rats with OC increases the plasma and hepatic TGF-β levels, which is thought to be caused by the estrogenic component [12], since E2 induces both the synthesis and secretion of TGF-β in thyroid stromal cells as well as Smad-2 phosphorylation via ER-α and ER-β [191]. Interestingly, although the plasma and liver TGF-β levels remain elevated, the paradoxical anti-fibrotic effects of OC may be caused by the dose, time, and even the contrasting responses due to the differential stimulation on both ERs regulating TGF-β activity [191,192]. Moreover, estrogens and their metabolites are also suggested as anti-fibrotic agents, which have little or no affinity for ERs, elicit divergent effects versus the progestins [183], and because of the bidirectional actions of TGF-β. Furthermore, despite the suggested pro-fibrotic effects of progestins, medroxyprogesterone acetate (MPA) inhibits TNF-α-induced matrix metalloproteinase (MMP)-9 via the glucocorticoid receptor [92]. MMP-9 has been proven as an important mediator of leukocyte recruitment and a target in liver injury because of its pro-fibrotic effects [193,194]. In this case, MPA induces an immunomodulatory effect by inhibiting TNF-α and may result in an anti-fibrotic activity in the liver, which shows, again, the dual behavior of these hormone analogs.

Regarding the PE effects on liver fibrosis by immunomodulation, the O-methylated isoflavone calycosin inhibits the expression of ECM proteins and the proliferation, activation, and migration of HSC induced by TGF-β1. This effect was correlated with calycosin binding and down-regulation of the ERβ5 subtype [195]. Genistein has also been shown to possess hepatoprotective effects in D-GalN-induced fulminant hepatic failure in rats by modulating the NF-κB/MAPK pathways [161] and anti-fibrotic effects in chronic damage by the Smad7-induced inhibition of TGF-β/Smad2/3 [196]. Here, the Smad superfamily is the main signal transducer for receptors of TGF-β during fibrosis [197]. BCA is a PE that was also evaluated as antifibrotic by tackling different molecular mechanisms: Both NF-κB and TGF-β1 were found up-regulated in fibrotic lesions, while BCA decreased their expression [149]. For a concise description of experimental and clinical evidence of beneficial, bidirectional, and deleterious effects elicited by female sex hormones, OC, and PE, please see Table 1.

## 5. Preclinical and Clinical Studies on the Effects of Sex Hormones or their Analogs in Specific Liver Diseases

### 5.1. HBV and HCV Chronic Infections: Oxidative Stress and Fibrosis as a Consequence

In experimental fibrogenesis models induced by dimethylnitrosamine (DMN), the use of estrogens has shown a protective effect [207]. Women infected with HCV develop more fibrosis in the postmenopausal stage than in the active reproductive phase, which could be due to the protecting effect of estrogen [189,208,209]. In human immunodeficiency viruses (HIV)/HCV-coinfected perimenopause women, confirmed by serum levels of anti-müllerian hormone (AMH), a greater progression of hepatic fibrosis was demonstrated [210]. This was related with an improvement in HCV replication compared to monoinfection of HCV, which potentiates and accelerates the development of fibrosis [211]. On the other hand, postmenopausal women with hormone replacement therapy have been shown to develop less liver fibrosis [189,208]. Likewise, similar data have been observed in nonalcoholic fatty liver disease in premenopausal women compared to postmenopausal women [212,213]. Finally, these data have also been consistent in studies in animals in which decreased collagen synthesis has been observed from stellate liver cells treated with E2 [207].

As aforementioned, the protective effect of estrogens in fibrosis occurs basically in relation to the reduced expression of procollagen type I and the tissue inhibitor of metalloproteinases (TIMP)-1, as well as a decrease in the deposition of hepatic type I and collagen type III [187]. It has been proposed that E2 suppresses the expression of smooth muscle actin (α-SMA), an activator of HSC, and restores the retinyl palmitate content in experimental liver fibrosis [214,215]. The mechanism by which E2 inhibits the function of HSC could be related to an extrathymic pathway of T cell differentiation in the liver [216] and an increase in IFN-γ activity [217]. It has also been shown that lipid peroxidation and acetaldehyde stimulate the expression of collagen, and that E2 and its derivatives inhibit lipid peroxidation of hepatic mitochondrial membranes induced by adenosine 5’-diphosphate (ADP) and FeSO_4_ [218]. Then, it has been suggested that E2 may have a suppressive effect on liver fibrosis through the stimulation of IFN and/or the inhibition of lipid peroxidation.

Also, the use of idoxifene, a tissue-specific selective ER modulator, and E2 inhibits IκB-α degradation and NF-κB activation by attenuating oxidative reactions and mitochondrial lipid peroxidation of hepatocytes [219]. In addition, treatment with idoxifene and E2 in the hepatic fibrosis model demonstrated the suppression of DMN-mediated necrosis and lipid peroxidation as well as the absence of antioxidant enzyme activity and proapoptotic status through the expression of Bcl-2 family proteins [184]. On the other hand, the use of catechol estrogens, metabolites derived from E2, were shown to have antioxidant effects at concentrations greater than 1 µM, inhibiting the lipid peroxidation induced by ferrylmyoglobin in rat hepatocytes. In contrast, at physiological concentrations (100 pM–100 nM), catechol estrogens had a pro-oxidant effect. In the thioacetamide-induced rat liver cirrhosis model, estrogen use improved the proportion of collagen, lipid peroxidation, and the expressions of alpha-SMA and STAP. Likewise, it has been proposed that treatment with E2 benzoate using this same experimental model induces cirrhosis, associated with increased oxidative stress and HSC stimulation, with no increase in the formation of 8-hydroxy-2-deoxyguanosine (8-OHdG) [182], in contrast to breast carcinoma cell lines.

### 5.2. Cholangitis and Cholestasis

Cholangiopathies include primary biliary cirrhosis and primary sclerosing cholangitis, which share features such as intralobular cholestasis, disappearance of interlobular bile ducts, and proliferation of residual bile ducts. In this regard, the proliferation of cholangioli is considered an adaptive phenomenon in the evolution of disease to ductopenia and the absence of damaged bile ducts [220,221]. Intrahepatic cholangiocytic proliferation in rats has been shown to be stimulated by estrogens. This mechanism of cholangiocyte proliferation has been associated with increased expressions of total and proline-rich receptor-like protein kinase (p-ERK1/2) and of the adapter protein Shc, with the inhibition in epithelial proliferation using ER antagonists in vivo (tamoxifen and Ici 182,780), as well as an increase in the expression of p-ERK1/2, Src, and Shc induced by 17β-E2 in vitro. Thus, it has been suggested that the estrogen-activated ER/Src/Shc/ERK signaling pathway could be involved in cholangiocytic proliferation, possibly acting synergistically with growth factors through tyrosine-kinase receptors. That is why one of the proposals for the treatment of cholangiopathies is the use of ER agonists to block cholangiocytic proliferation, and the use of MAPK inhibitors or coactivators (Src, Shc) has also been proposed in neoplastic processes such as cholangiocarcinoma or dysplastic disorders (polycystic liver disease) [222].

Through a bile duct ligation model, it was shown that liver enzyme levels were significantly inferior in those animals with the lowest estrogen levels. Likewise, the changes associated with hepatocellular damage such as necrosis, ductular proliferation, Kupffer cell abnormalities, and sinusoidal congestion were less severe. Also, in this study it was observed that IL-6 was significantly lower, while IFN-γ was higher, in association with low serum estrogen levels, which correlated with decreased regeneration and fibrogenesis [93]. In chronic hepatitis C, the release of TNF-α and IL-1β by circulating mononuclear cells is greater than in healthy individuals. In these cases, E2 has been shown to attenuate the production of IL-1β in ERs expressing hepatoblastoma HepG2 cells [223]. On the other hand, in vivo, transdermal administration of E2 in postmenopausal women has been found to decrease the production of IL-6 [224].

### 5.3. Non-Alcoholic Fatty Liver Disease (NAFLD) and Non-Alcoholic Steatohepatitis (NASH): Gender Differences

NAFLD leads to NASH, fibrosis, and cirrhosis [225]. Liver fibrosis has been linked to the production of estrogen and P4 [226,227] as well as reproductive system dysfunction. In experimental models in male rats with hepatic fibrosis induced by CCl_4_, associations with testicular degeneration, hydrocele, fibrosis, scarring, calcifications, hypogonadism, and cryptorchidism have been observed. Then, changes in testicular function, impaired fertility, decreased pregnancy rate, and impaired development of the gonad have been related to liver fibrosis [228]. In aromatase-deficient mice, unable to produce estrogens and suffering from deterioration of the hepatocellular fatty acid β-oxidation, hepatic steatosis develops spontaneously. Also, in E2 replacement therapy the liver is reduced, and the mitochondrial and peroxisomal fatty acid β-oxidation is restored. It is likely that the progression of liver injury with steatosis in males may be due to the decrease in E2 production and/or the lower response to its actions [147].

### 5.4. Hepatocellular Carcinoma (HCC)

Estrogens and ERs have been associated with the clinical evaluation of chronic liver disease. The induction of lipid peroxidation, functional alterations of superoxide dismutase, and development of hepatocellular carcinoma are considered secondary effects to the decrease of ERs in the menopause stage [229]. It is known that synthetic and natural estrogens increase the incidence of liver tumors in response to their tumor-promoting activity [230]. Apparently, the mechanisms involved in this are related to alterations in liver function secondary to interactions with ER, and it is also possible that the effect is associated with a mitogenic hepatocyte activator [74,231,232]. Likewise, it has been proposed that molecular signaling pathways involved in cell growth and differentiation could be related to ER promoter activity [233]. In this regard, estrogens have been demonstrated as modulators of uterine EGFRs in rat models [234].

In experimental models of hepatocarcinogenesis induced by diethynylnitrosamine (DEN), an initiating agent, and EE as a promoter, an incidence of neoplasms of about 80% was found. The most relevant changes associated with this study revealed an increase in markers of cholestasis, increased ER receptor concentration and occupation, increased cell proliferation, and metabolic activation of E2 and EE [105]. Another mechanism related to liver tumorigenesis has been related to the stimulation of DNA synthesis secondary to synergistic events between EGF and 17β-E2 in primary cultures of rat hepatocytes. Apparently, this could be associated with c-fos RNAm expression; however, it is necessary to elucidate the specific mechanisms [235]. In contrast, one study demonstrated the suppressive effect of E2 on rat chemical hepatocarcinogenesis induced by DEN/2-acetylaminofluorene (AAF)/partial hepatectomy [236].

It is well known that chronic HBV infection is the most common cause of cirrhosis and hepatocellular carcinoma. Also, statistically, chronic hepatitis B and C tend to progress more rapidly in men than in women, and liver cirrhosis is more frequent in men and in postmenopausal women [237,238]. In addition to E2 inhibiting ROS generation and its antioxidant activity through the suppression of NADH/NADPH oxidase activity, blocked expression of hydrogen peroxide-induced TGF-β and the activation of AP-1 and NF-κB, which facilitates carcinogenesis, have been reported [135]. Therefore, it has been suggested that, in treatments that suppress NADH/NADPH oxidase activity, E2 could have a cytoprotective effect on the development of hepatocellular injury.

## 6. Conclusions

The liver is a very important organ that impacts endocrine and immune responses, and vice versa, and the sex hormones, OC, and PE have immunomodulatory activities that mediate beneficial and deleterious processes in both health and disease. By analyzing the current data, mostly obtained from animal experiments and cell culture, it can be observed that such effects depend on concentration, dose, time of administration, the particular drug or mixture ingested, and the hepatic injury model used. Also, it is perceived that clinical studies are urgently needed to define the potential therapeutic uses of these drugs for specific liver diseases, such as fibrosis and MetS, or, on the contrary, to avoid their intake during acute or chronic hepatic illnesses. This can help reduce the worsening of cholestasis and cholangiopathies, all of which come along with oxidative stress.

## Figures and Tables

**Table 1:** Effects of female sex hormones, oral contraceptives, and phytoestrogens on the liver.

Compound	Effect	Immunomodulation and Biochemical Mechanisms	Doses/Concentration	Ref.
**Estrogens and combined OC** **(a) Deleterious effects:**				
OC	Cholestasis. Oxidative stress (mitochondrial, and endoplasmic reticulum) in endotoxin-induced liver injury in rodents	Activation of Kupffer cells and production of TNF-α, IL-6, and IL-1β	Female Sprague-Dawley rats: 20 mg/kg Female Wistar rats: 17 alpha-Ethinylestradiol (EE) 35 ng/kg and Norethindrone 2 μ/kg	[111,112,113,114,198]
E2	Enhances cirrhosis induced by thioacetamide in rats	Accumulation of hepatic collagen, LP level, collagen amount, and cirrhosis degree	F344 rats: 100 µg/kg	[24,98,182]
**(b) Beneficial effects:**				
E2	Reduces fibrosis in various liver injury models in ovariectomized rats	Decreases TGF-β production, collagen synthesis, and oxidative stress, as well as the MAPK pathways	Female Sprague Dawley: 20 μg/kg/day	[183,186]
OC	Reduces liver fibrosis in vitro, female rats or in women	Inhibits collagen synthesis, despite elevated plasma and hepatic TGF-β levels. Inactivates the downstream transcription cascade of TGF-β1 expression and HSC activation.	Wistar rats: 200 μ/kg	[12,135,142,143,165,187,199,200,201,202]
	High cardiometabolic risk during intake (reversible)	Increase of hepatic glycogen stores in normal rats	Norgestrel and 20 µ/kg EE	
	5841 women (age range 24–49 years) from three population-based cohorts. Women using OC or POCs. Metabolomic profiles were reassessed for 869 women after six years	Increases triglycerides, HDL, Apo C and A-I, insulin, PCR, and SHBG; decreases testosterone; changes fatty acids (decreased ω-6 and increased monounsaturated and saturated) and amino acids (increased Phe and decreased Tyr levels); reduces albumin levels but increases creatinine, glycoprotein acetyls, growth factors (SCGFβ, βNGF, SCF, VEGF, FGF, PDGF-BB), and IL-2rα, IL-12p70, and IL-17. Cytokines IL1β, IL-6, and TNF-α displayed weak and non-significant associations	30–40 μg EE	
OC, E2 and EE	Hepatoprotective in normal and in diabetic models in mice	Increase of the hepatic glycogen stores in normal rats, lowering the plasma glucose	Female albino mice: 5 µg/kg of 17 beta βE2, 5 µ/kg of EE, 1 mg/kg of P4, 1 mg/kg of norethisterone acetate	[142,144,199]
EE and DRSP	Continuous administration of EE and DRSP leads to hyperinsulinemia in female mice	Augmentation of glucose-induced insulin secretion, decreased insulin clearance, and reduced IRβ protein expression in the liver	/	/
**(c) Bidirectional effects:**				
OC (EE and norgestrel)	Chronic cholestasis in Wistar rats	High LP levels, cytokine imbalance, increased plasma TNF-α and IL-10 in the liver, as well as TGF-β	Wistar rats: 100, 200 μg/kg norgestrel, 10, 20 μg/kg EE/14 and 28 days	[12,127,129,133]
	209 Women aged 40–48 years who used OC, compared to non-users and intrauterine users of hormonal and copper devices	Augmentation of LP, antioxidants in plasma were diminished, LDLs elevated while β-carotene and zinc were lowered	Combined OC: EE (0.020–0.035 mg + Progestine (0.075–3.0 mg). IUD (LNG 0.02 mg)	
	32 women 18–35 years old who consumed the OC combination of EE and drospirenone	Combinations of OC inhibit liver PON activity, whilst serum PON activity is augmented; CAT activity in erythrocytes was reduced by all OCs tried. Estrogens (primarily E2) possess antioxidant effects	0.03 mg of EE and 3 mg drospirenone	
	OC in mice	Diminishing ROS, activation of NF-κB and AP-1, pro-inflammatory cytokines. Up-regulates antioxidant enzymes and expression of the Bcl-2 family proteins.	0.15 mg Desogestrel + 0.03 mg EE; 0.15 mg LNG + 0.03 mg EE and 0.15 mg desogestrel+0.02 mg EE / 21 days each drug	
		Diminished LP and TNF-α-induced apoptosis.		
**Progestins** **(a) Deleterious effects:**				
P4 and its metabolites	Intrahepatic cholestasis of pregnancy in isolated perfused rat liver	High quantities of PM4, PM4-Sul, and epiallopregnanolone-sulfate (PM5-Sul) in the urine of IPC patients has been correlated with failure of biliary canalicular transporters (BSEP, MRP-2, and MRP-3)	3 µmol to the recirculation media used to in situ perfuse the rat liver	[125,126]
P4 and norgestrel	Prooxidant and pro-inflammatory effects in murine	Oxidative stress and LP, production of TNF-α, IL-1β, MIP-2, and MCP-1	/	[134,135]
	peritoneal macrophages and cultured rat hepatic stellate cells	Generation of ROS, TGF-β in activated HSC		
P4	Mononuclear cells from patients with chronic hepatitis C	Production of TNF-α, IL-1β, IL-8, and macrophage chemotactic protein (MCP)-1	10^−7^ mol/L	[94]
**(b) Beneficial effects:**				
MPA	Anti-inflammatory effects in epithelial cells.	Inhibits the TNF-α-induced matrix metalloproteinase (MMP)-9 via the glucocorticoid receptor	10^−6^ M/ 72 h.	[92]
**Phytoestrogens** **(a) Deleterious effects:**				
Miroestrol and deoxymiroestrol	Intrahepatic cholestasis in C57BL/6 mice.	Regulates the transporter expression (BSEP and MRP2 mRNA in both male and female mice), though the immunomodulatory effects is not known.	0.5 mg/kg/day once a day for 7 days	[124]
**(b) Beneficial effects:**				
Resveratrol and ginsenosides	Prophylactic against EE-induced liver cholestasis	Reduced marker levels of cholestasis, oxidative stress, as well as of the pro-inflammatory cytokines TNF-α, IL-6, and IL-1β	25 mg/kg/15 days of resveratrol and 30–300 mg/kg of gingenosides, i.g. /5 days	[123]
Resveratrol	Protects against atherosclerosis and hepatic steatosis in vitro and in vivo	Down-regulation of SREBP-1c expression through the ERα-mediated pathway in L02cells. Induces a mitochondrial complex I-dependent increase in NADH oxidation resulting in sirtuin activation in HepG2 cells and in mice	In LO2 cells: 10–20 µM HepG2 cells: 1–5 µM. C57BL6/N: 50 mg/kg/day	[153,154]
Biochanin A	Protects against acute CCl_4_-induced hepatotoxicity in Wistar rats	Protects from oxidative stress measured by LPO, GSH, SOD, total antioxidant total, catalase activities, and inhibition of iNOS, COX2, and CD45 expression	Wistar: 25–1600 mg/kg; and 50 mg/kg	[149,151,152,203]
	Antifibrotic effect in rats.	Decreased the expressions of NF-κB, TGF-β1, MMP9, TNFα	Male Sprague-Dawley with diabetes: 10–40 mg/kg/28 days	
	Improves insulin sensitivity, hepatic steatosis, and controls hyperglycemia in type 2 diabetes and obesity models	Antifibrotic effects by decreasing the expressions of NF-κB, TGF-β1, MMP9, and TNFα.		
	BCA protected against LPS/GalN-induced acute liver injury in mice	Activating the Nrf2 pathway and inhibiting NLRP3 inflammasome activation		
	Improve type 2 diabetes induced in rats	BCA improves insulin sensitivity and increases the expression of SIRT1 histone deacetylase in pancreatic tissue in induced type 2 diabetes	In C57BL/6 mice with obesity: 0.05%/12 weeks (wk)	
	Beneficial effects on obesity-mediated hepatic steatosis and insulin resistance of obese mice	BCA increases the expression of PPAR-α and its regulatory proteins in the liver.		
Formononetin and biochanin A	Protection against ritonavir induced hepatotoxicity in adult male Sprague–Dawley rats	Hepatoprotection via modulation of oxidative stress, inflammation, and apoptosis: NFkB/pAkt signaling molecules, caspase-3, NFκB, and eNOS activation	100 mg/kg, p.o.	[150]
Genistein	Inhibition of HCC in mice (C57BL/6 N) treated with DEN at 2 weeks of age and fed with supplemental of genistein	Increase of phospho-AMPK in the liver, Hep3B, and Raw 264.7 cells. Inhibition of NF-κB levels, and down-regulation of TNF and IL-6	C57BL/6 N: 80 mg/kg/day, for 5 months, from 40 to 62 wk of age	[158,159,160,162,163,196,197,204]
	Ameliorate NAFLD in C57BL/6 mice, Hep-G2 cells	Inhibition of COX-1 activity as well as its downstream TXA_2_ biosynthesis	C57BL/6 mice: 1.5–64 mg/kg for 22 wk Hep-G2: 100 μM	
	Improved NAFDL in high-fat/high-sucrose diet-treated Sprague–Dawley rats.	In steatosis hepatic via AMPK, thus promoting fatty acid oxidation and inhibiting hepatic lipid synthesis (mRNA levels of FAS and GPAT were lower, but PPARα, CPT-1, and ACO were higher in rats treated with genistein)	Sprague-Dawley rats: 4–8 mg/kg body weight	
	Hepatoprotective and anti-fibrotic effects in D-galactosamine (D-GalN)-induced fulminant hepatic failure in Wistar rats	Decreased AST and ALT and increased iNOS, COX-2, NO, and PGE. Suppression of TNF-α, IL-1β, NF-κB, IKKα/β, and MAPK phosphorylation	5 mg/kg BW/day/30 days, i.g.	
	Alleviates hepatic damage induced by chronic alcohol in mice	Decreasing levels of MDA, TNF-α, IL-6, ALT, and LDL.	/	
		Inhibition of iNOS, TNF-α, NF-ĸB, and caspases-3	0.3 mmol/kg with 50% alcohol once per day for 5 weeks.	
	Attenuates DGalN-induced liver fibrosis/chronic liver damage in rats	Hepatoprotection by modulating the NF-κB/MAPK pathways and chronic damage by the Smad7-induced inhibition of TGF-β/Smad2/3	5 mg/kg BW i.g./12 wk	
Daidzein	Modulate hepatic glucose and lipid-regulating enzyme activities in C57BL/KsJ-db/db mice	Decrease in blood glucose and HbA(1c) levels, increased the insulin/glucagon ratio in the type 2 diabetic animals	C57BL/KsJ-db/db mice: 0.02% *w/w*	[166,167]
	Potent inducer of apoptosis in hepatic cancer cells (SK-HEP-1)	Apoptosis associated with the up-regulation of Bak and down-regulation of Bcl-2 and Bcl-xL proteins, caspases 3 and 9 in SK-HEP-1 cells	200 μM, 400 μM, or 600 μM	
Coumestrol	Increase in mitochondria number and function in cultured skeletal muscle cells (C2C12).	Activation of SIRT1, ATP levels, glucose uptake, and the protein expression of respiratory chain components. Stimulation of mitochondrial biogenesis.	5–10 μM	[205,206]
Xanthohumol (XN) and 8-prenylnaringenin (8PN)	Ameliorated diabetic-related metabolic dysfunctions in C57Bl/6 mice during 20 weeks.	Promote hepatic and skeletal muscle AMP-activated protein kinase (AMPK), diminishing the expression of target lipogenic enzymes: SREBP-1c, FAS, and acetyl-CoA carboxylase activity. Moreover, both XN and 8PN treatments decreased the VEGFR-1/VEGFB pathway.	C57Bl/6 mice: 10 mg/L of XN and 8PN.	[171,172,173,174]
	In apolipoprotein-E-deficient (ApoE^−/−^) mice fed a Western-type diet reducing hepatic lipogenesis.	Induction of SREBP-1c mRNA and CPT-1a or increased fatty acid beta-oxidation.	Female ApoE^−/−^ mice: 300 mg/kg body weight/day for 8 wk	
	In vitro: platelet aggregation.	Inhibition of NF-κB, TGF-β1 and IL8	1.5 And 3 μM in platelet assay	
	In female BALB/c mice with induced NASH	Prevent body weight gain; decreased glycemia, triglycerides, cholesterol, and alkaline phosphatase levels; improved insulin sensitivity in mice, thus suppressing lipogenesis	Female BALB/c: Diet with 1% XN w/w for 3 wk	
	Primary human hepatocytes (PHHs)	XN attenuates atherosclerosis	PHH: 25 and 50 μM	
Calycosin (*O*-methylated isoflavone)	Anti-fibrotic activity in activated HSCs	Inhibition effect on expression of migration, proliferation, activation, and migration of HSC induced by TGF-β1	182–780 μM	[195]
**(b) Bidirectional effects:**				
Enterolactone	Development of hepatic insulin resistance and enhanced apoptosis in HepG2 cells	Increase in COX-2 and TNFα protein expressions, resistance of insulin and apoptosis (caspase 3), and stimulation of the de novo ceramide synthesis pathway	50 μM of enteronolactone and with palmitic acid 0.5 mM	[169]

^1^ Please see the glossary for abbreviations.

## Abbreviations

AAF	2-Acetylaminofluorene
Ab	Antibody
AHR	Aryl hydrocarbon receptor
AKT	Kinase/protein kinase B
AMH	Anti-müllerian hormone
AMPK	AMP-Activated protein kinase
AP-1	Activator protein-1
APC	Antigen-presenting cell
APR	Acute phase reactants
ATP	Adenosine triphosphate
ATP5a	ATP synthase mitochondria F1 complex α subunit 1
BA	Bile acid(s)
BCA	Biochanin
BSEP	Bile salt export pump
BSF-2	B cell stimulatory factor 2
cAMP	Cyclic adenosine monophosphate
CBG	Corticosteroid-binding globulin
CCL20	C-C Chemokine ligand 20
CCND1	Cyclin D1
CCR4	C-C Chemokine receptor type 4
CCR6	C-C Chemokine receptor type 6
CD	Cluster of differentiation
CD4+	CD4+ T lymphocytes
CD8+	CD8+ T lymphocytes
Cdk	Cyclin-dependent kinase
CEBP-β	CCAAT/enhancer-binding protein-β
cGMP	Cyclic guanosine monophosphate
CNTF	Cilliary neurotrophic factor
COX	Cyclooxygenase
CRP	C-reactive protein
CSF	Colony-stimulating factor
CTLA-4	Cytotoxic T-lymphocyte-associated antigen-4
CU	Coumestrol
Cxcl	Chemokine C-X-C motif ligand
DA	Daidzein
DEN	Diethynylnitrosamine
DNA	Deoxyribonucleic acid
E1	Estrone
E2-17G	Estradiol-17β-glucuronide
E2V	E2 valerate
ECM	Extracellular matrix
EE	17α-ethinylestradiol
EGF	Epidermal growth factor
EGFR	Epidermal growth factor receptor
ENL	Enteronolactone
ER	Estrogen receptor(s)
ERE	Estrogen response element
ERK	Extracellular signal-regulated kinase
FAS	Fatty acid synthase
FAT	Fatty acid influx
FDA	Food and Drug Administration
FGF	Fibroblast growth factor
FoxP3	Forkhead transcription factor
FSH	Follicle-stimulating hormone
GE	Genistein
GF	Growth factor
GITR	Glucocorticoid-induced TNF-alpha receptor
GnRH	Gonadotropin-releasing hormone
GPCR	G protein coupled receptor
GPER/GPR 30	G protein-coupled estrogenic receptor
GR	Glucocorticoid receptor
Grb2	Growth-factor-receptor-bound protein 2
GSH	Reduced glutathione
GTF	General transcription Factor
HB-EGF	Heparin-binding-EGF
HBV-LF	Hepatitis B virus-related liver fibrosis
HCC	Hepatocellular carcinoma
HCV	Hepatitis C virus
HDL	High-density lipoproteins
HGF	Hepatocyte growth factor
HIV	Human immunodeficiency viruses
HRE	Hormone response element
HSC	Hepatic stellate cells
HSP	Heat shock protein
ICP	Intrahepatic cholestasis of pregnancy
IFN	Interferon(s)
IGF-1	Insulin-like growth factor 1
IL	Interleukin(s)
iNOS	Inducible nitric oxide synthase
JAK	Janus kinase
LBD	Ligand-binding-domain
LDA	Ligand dependent pathway of activation
LDL	Low density lipoproteins
LH	Luteinizing hormone
LIF	Leukemia inhibitory factor
LNG	Levonorgestrel
LP	Lipid peroxidation
M	Macrophage(s)
MAP	Mitogen-activated protein
MAPK	Mitogen activated protein kinase
MCP	Macrophage chemotactic protein
MetS	Metabolic syndrome
mPR	Membrane localized progestin receptor
MRP	Multidrug resistance-associated protein
MyD88	Myeloid differentiation primary response protein 88
NAFLD	Non-alcoholic fatty liver disease
NASH	Non-alcoholic steatohepatitis
NDUFA9	NADH dehydrogenase 1α subcomplex
NF	Nuclear factor
βNGF	β-Nerve growth factor
NHR	Nuclear hormone receptors
NK	Natural killer
NLRP	NOD-like receptor
NO	Nitric oxide
Nrf2	Nuclear factor erythroid 2
NT	Neurotransmitter
NTCP	Na+-taurocholate cotransporter protein
OATP	Organic anion transporting protein
OC	Oral contraceptive(s)
O-DMA	O-demethylangiotensin
OVX	Ovariectomy
P4	Progesterone
PDGF-BB	Platelet-Derived Growth Factor-BB
PE	Phytoestrogen(s)
PELP1/MNAR	Proline-, glutamic acid-, and leucine-rich protein 1, modulator of non-genomic activity of ERs
p-ERK1	Proline-rich receptor-like protein kinase
PI3K	Phosphoinositide 3-kinase
PKA	Protein kinase A
PKC	Protein kinase C
PON	Paraoxonase
PPAR-γ	Peroxisome proliferator-activated receptor-gamma
8PN	8-Prenylnaringenin
PR	Progestin receptor
PRL	Prolactin
Ras	Small GTPase named by abbreviating rat sarcoma
ROS	Reactive oxygen species
SCF	Stem cell factor
SCGF-β	Stem cell growth factor beta
SDHA	Succinate dehydrogenase complex subunit A
Sgk	Serum and glucocorticoid regulated kinase
SGK	Glucocorticoid induced kinase
SHBG	Sex hormone-binding globulin
SHP	Protein tyrosine phosphatase
sIL-6R	Soluble derivative of the IL-6 receptor
SIRT	Silent mating type information regulation 2 homolog (Sirtuin) NAD^+^-dependent protein deacetylase
SOCS	Suppressor of cytokine signaling
SOD	Superoxide dismutase
Sos	Protein son-of-sevenless
Src	Schmidt-Ruppin A-2 viral oncogene tyrosine-protein kinase
SREBP-1c	Sterol regulatory element binding protein-1c
STAT3	Signal transducer and activator of transcription factor-3
TCR	T cell receptor
TF	Transcription factor
TGF	Transforming growth factor
Th	T helper cell(s)
TLR	Toll-like receptor
TNF	Tumor necrosis factor
Treg	T regulatory cell(s)
TX	Tromboxane
TYK	Phosphorylated tyrosine Y
UQCRC2	Ubiquinol cytochrome c reductase core protein
VEGF	Vascular endothelial growth factor
VLDL	Very low density lipoprotein
XN	Xanthohumol
